# Nelson Bay Reovirus Isolated from Bats and Blood-Sucking Arthropods Collected in Yunnan Province, China

**DOI:** 10.1128/spectrum.05122-22

**Published:** 2023-06-12

**Authors:** Guopeng Kuang, Ziqian Xu, Jing Wang, Zhangjin Gao, Weihong Yang, Weichen Wu, Guodong Liang, Mang Shi, Yun Feng

**Affiliations:** a Yunnan Provincial Key Laboratory for Zoonosis Control and Prevention, Yunnan Institute of Endemic Disease Control and Prevention, Dali, China; b State Key Laboratory of Infectious Disease Prevention and Control, National Institute for Viral Disease Control and Prevention, Chinese Center for Disease Control and Prevention, Beijing, China; c Centre for Infection and Immunity Studies, School of Medicine, Shenzhen Campus of Sun Yat-sen University, Sun Yat-sen University, Shenzhen, China; d School of Public Health, Dali University, Dali, China; e College of Global Change and Earth System Science, Beijing Normal University, Beijing, China; Centro de Investigacion y de Estudios Avanzados del Instituto Politecnico Nacional

**Keywords:** Nelson Bay reovirus, bat, bat fly, vector, metatranscriptomics, genomic reassortment

## Abstract

Nelson Bay reovirus (NBV) is an emerging zoonotic virus that can cause acute respiratory disease in humans. These viruses are mainly discovered in Oceania, Africa, and Asia, and bats have been identified as their main animal reservoir. However, despite recent expansion of diversity for NBVs, the transmission dynamics and evolutionary history of NBVs are still unclear. This study successfully isolated two NBV strains (MLBC1302 and MLBC1313) from blood-sucking bat fly specimens (*Eucampsipoda sundaica*) and one (WDBP1716) from the spleen specimen of a fruit bat (Rousettus leschenaultii), which were collected at the China-Myanmar border area of Yunnan Province. Syncytia cytopathic effects (CPE) were observed in BHK-21 and Vero E6 cells infected with the three strains at 48 h postinfection. Electron micrographs of ultrathin sections showed numerous spherical virions with a diameter of approximately 70 nm in the cytoplasm of infected cells. The complete genome nucleotide sequence of the viruses was determined by metatranscriptomic sequencing of infected cells. Phylogenetic analysis demonstrated that the novel strains were closely related to Cangyuan orthoreovirus, Melaka orthoreovirus, and human-infecting Pteropine orthoreovirus HK23629/07. Simplot analysis revealed the strains originated from complex genomic reassortment among different NBVs, suggesting the viruses experienced a high reassortment rate. In addition, strains successfully isolated from bat flies also implied that blood-sucking arthropods might serve as potential transmission vectors.

**IMPORTANCE** Bats are the reservoir of many viral pathogens with strong pathogenicity, including NBVs. Nevertheless, it is unclear whether arthropod vectors are involved in transmitting NBVs. In this study, we successfully isolated two NBV strains from bat flies collected from the body surface of bats, which implies that they may be vectors for virus transmission between bats. While the potential threat to humans remains to be determined, evolutionary analyses involving different segments revealed that the novel strains had complex reassortment histories, with S1, S2, and M1 segments highly similar to human pathogens. Further experiments are required to determine whether more NBVs are vectored by bat flies, their potential threat to humans, and transmission dynamics.

## INTRODUCTION

*Orthoreovirus*, which belongs to the oder *Reovirales* and family *Spinareoviridae*, is a genus of nonenveloped viruses containing 10 double-strand RNA segments, including three large segments (L1, L2, and L3), three medium segments (M1, M2, and M3), and four small segments (S1, S2, S3, and S4) (1). Based on the ability to cause syncytial cytopathic effects (CPE) in infected cells, the orthoreoviruses can be further divided into the fusogenic and the nonfusogenic subgroups (2–4). The prototypical mammalian reoviruses are nonfusogenic and represent a phylogenetic clade distinct from the fusogenic reoviruses, such as Nelson Bay virus, avian reovirus, and baboon reovirus (4, 5). As the number of viral species increases within this genus, they were now divided into five major groups based on the phylogenetic relationships and host animals: namely, the mammalian orthoreoviruses (MRVs), which infect mammals, the avian orthoreoviruses (ARVs) infecting avians, Nelson Bay virus (NBV) and related orthoreoviruses infecting bats, baboon orthoreovirus infecting baboons, and the reptilian orthoreoviruses (RRVs) infecting reptilians (5). Furthermore, a sixth group, the piscine orthoreoviruses (PRVs), was recently proposed for the piscine hosts (6). Of all these viral species, only members of MRV and NBV can infect and cause diseases in humans.

In 1968, the first NBV was isolated from the blood of a fruit bat (*Pteropus poliocephalus*) in New South Wales, Australia, and was found to be lethal to mice (7). Since the discovery of NBV, more and more strains have been discovered in Asia and Africa. Among these, Pulau reovirus (8), Xi River reovirus (9), Cangyuan orthoreovirus (10), and Kasama virus (11) have been discovered only in bats, Pteropine orthoreovirus was discovered in both bats (12, 13) and monkey feces (14), and Melaka orthoreovirus (15), Kampar orthoreovirus (16), and Pteropine orthoreovirus (17–21) were discovered in patients with respiratory or enteric diseases. Epidemiological investigation of these cases suggests that these human-associated NBVs can be transmitted among humans (15, 20, 21), and the causative viruses may be bat-originated because some patients were exposed to bats prior to disease onset (15, 16). Therefore, NBV is a virus group with frequent cross-species transmission and relatively high zoonotic potentials.

Owing to the properties of segmented genomes, it seems not uncommon for the occurrence of genomic reassortment between orthoreoviruses. As a class of viruses commonly infecting humans, MRV has been widely studied, and reassortment events between different strains have been reported previously (22–27), as well as among ARV (28–31). Based on the difference in clustering and sequence homology of segments, genomic reassortment has also been found in the NBV group, such as Cangyuan orthoreovirus isolated from fruit bats in China (10) and Pteropine orthoreovirus HK23629/07, HK46886/09, and HK50842/10 from three patients whose exposures were all tied to travel to Indonesia (19). Diverse novel reovirus reassortants have been discovered, reflecting the role of reassortment events as a driving force in the emergence of new virus variants.

While NBVs are commonly known to infect bats, it is unclear how they transmit among hosts. It is widely believed that reoviruses primarily disseminate to every organ and tissue systemically within their mammalian hosts via the blood after replicating at the portal of entry (32–34). It is worth noting from the previous study that there are a variety of blood-sucking arthropods parasitized on the body surface of bats, and they can infect and spread viruses among bats through feeding behavior (35). Nevertheless, it has not been investigated whether any of the ectoparasites can transmit NBVs or related viruses. Indeed, it remains unclear that any member of the genus *Orthoreovirus* is a vector-borne pathogen. Accordingly, we investigated NBVs carried by bats and bat flies in some areas along the China-Myanmar border in Yunnan Province; further experimental studies were carried out to explore the intermediate hosts and transmission dynamics of these frequent reassortant viruses.

## RESULTS

### Host species identification.

The taxonomies of bats caught in this study were initially identified based on morphological traits. To further confirm the initial identifications, we analyzed the cytochrome c oxidase subunit I (*COI*) gene of the three specimens that showed syncytial CPE in virus isolation. The sequences of the *COI* gene were compared against the BOLD database, and it revealed that bat specimen WDBP1716 was likely to be Rousettus leschenaultii (99.85%), whereas bat fly specimens MLBC1302 and MLBC1313 were *Eucampsipoda sundaica* (99.42%), and these findings were consistent with morphological identifications.

### Virus isolation and identification.

**FIG 1:**
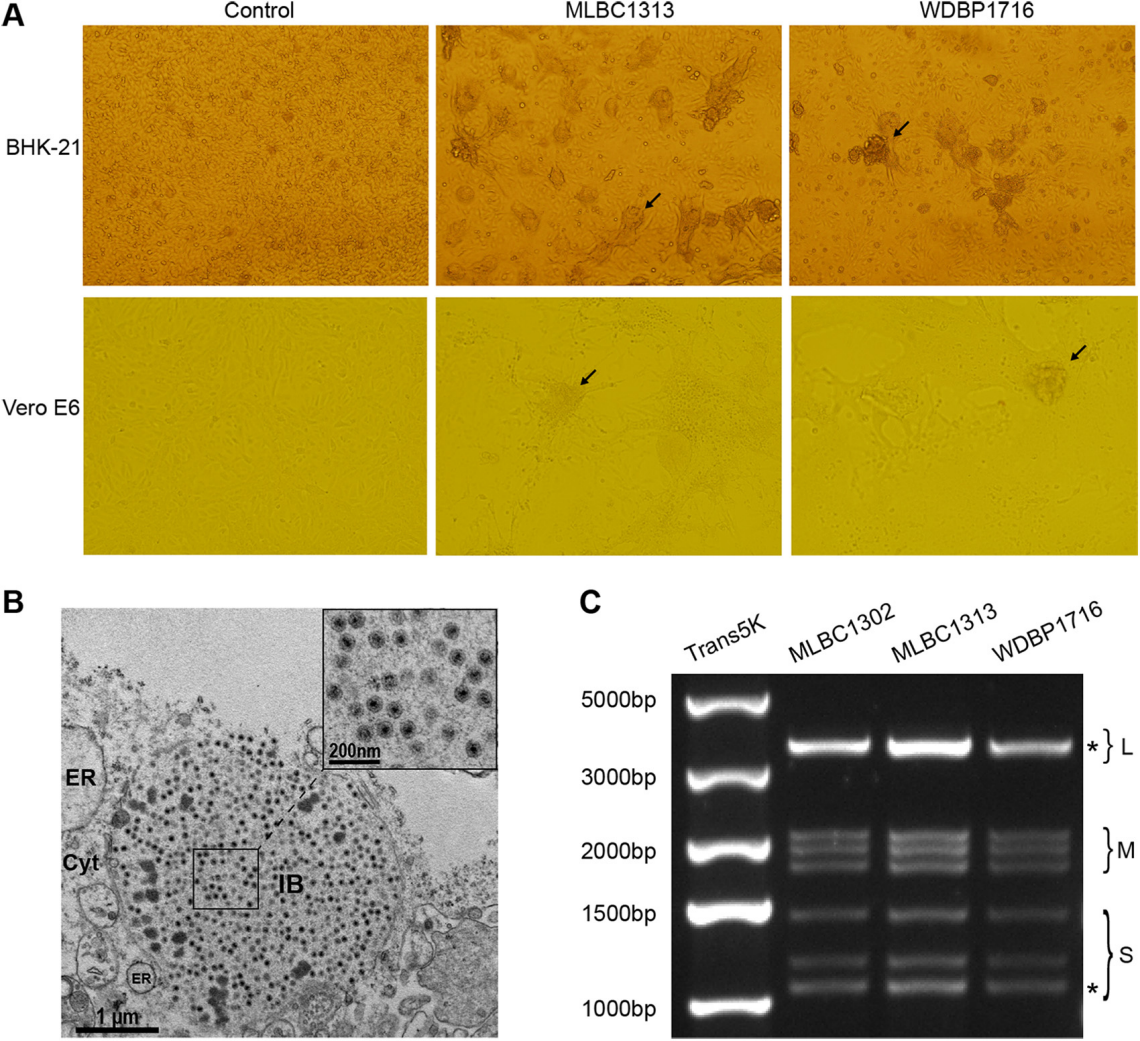
Isolation and identification of viruses. (A) Syncytial cytopathic effects (indicated with arrows) appeared in BHK-21 and Vero E6 cells inoculated with samples MLBC1313 and WDBP1716 at 48 h postinfection. (B) Electron micrographs of ultrathin sections show an inclusion body (IB) full of plenty of spherical virions in the cytoplasm (Cyt) of Vero E6 cells after inoculating with strain MLBC1313. Inset indicates large view of virons in IB. ER, endoplasmic reticulum. (C) Electropherotype for genome segments separated on a 1% agarose gel of the three strains, showing comigrating bands where more than one segment is indicated with asterisks.

Both bat spleen and bat fly specimens were subjected to virus isolation using monolayers of Baby hamster kidney (BHK-21) and African green monkey kidney (Vero E6) cells. The pools inoculated with strains WDBP1716 (bat), MLBC1302 (bat fly), and MLBC1313 (bat fly) showed CPE in both cell lines and formed multinucleated cellular syncytia at 48 h postinfection (Fig. 1A). The first-passage cells of three pools that showed CPE were inoculated again, and CPE was still observed in the second- and third-passage cells, which were considered positive for virus isolation. However, no CPE was observed after three passages in mosquito-derived C6/C36 cells, and the viral abundance levels, measured by qRT-PCR, decreased with each passage (Table S1), suggesting lack of growth in C6/C36 cell lines.

Electron micrographs for ultrathin sections were obtained after the third passage of MLBC1313 on Vero E6 cells, which showed an inclusion body (IB) full of spherical virions with a diameter of approximately 70 nm in the cytoplasm (Cyt) of infected cells (Fig. 1B). The same results were observed in cells infected by WDBP1716 and MLBC1302, indicating that the three novel strains isolated from bat and bat flies can also infect and actively replicate in mammalian cell lines.

Viral RNA was extracted from BHK-21 cells after three passages. Gel electrophoresis of total RNA showed similar electropherotype with Pulau reovirus and Melaka orthoreovirus (16) on a 1% agarose gel (Fig. 1C), which implied that they had similar genomic compositions.

Collectively, the three novel isolations were tentatively identified as species of the genus *Orthoreovirus* based on the CPE characters, virion morphologies, and the compositions of genome segments.

### Virus genome characterization.

**FIG 2:**
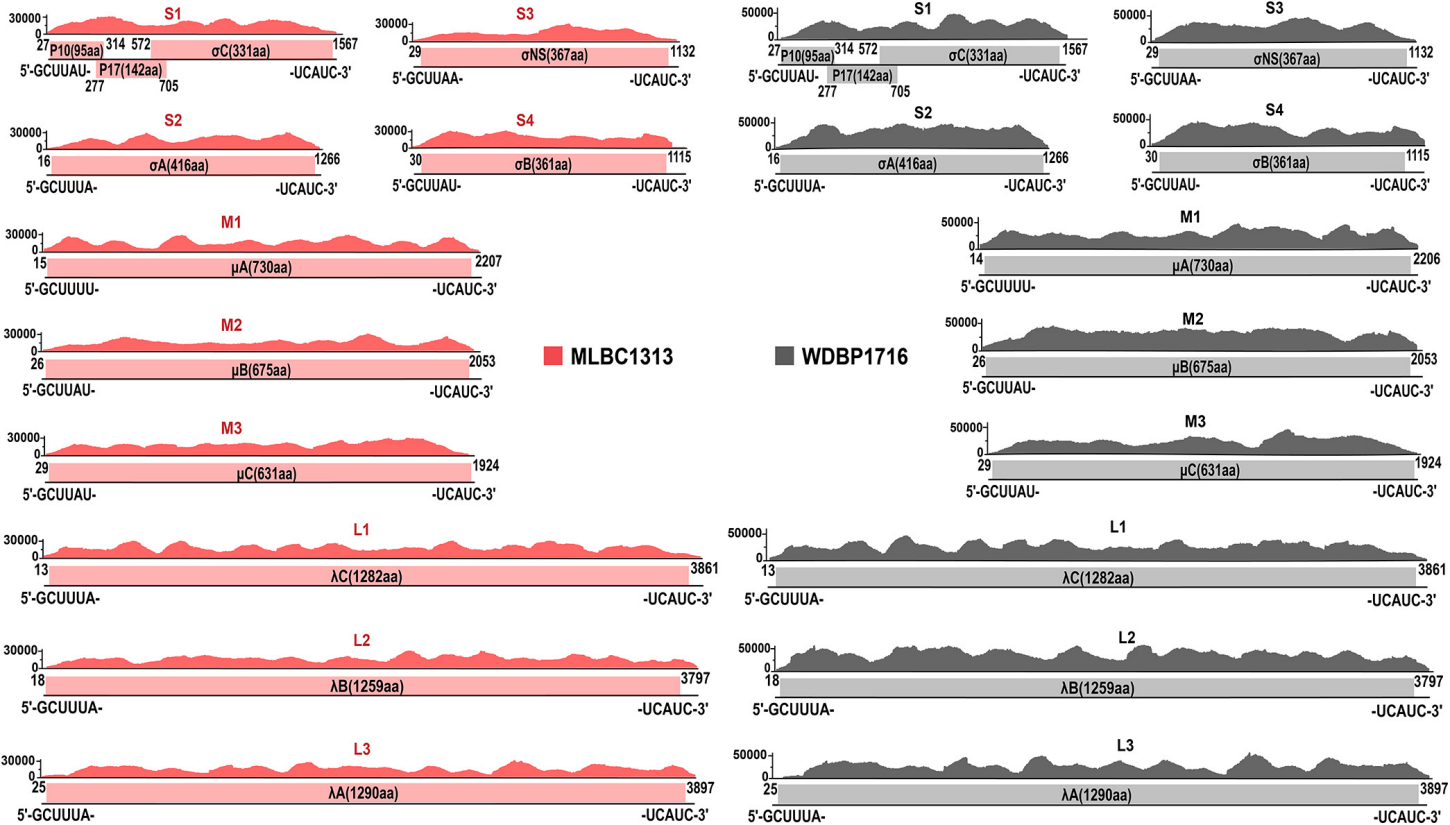
Sequencing coverage and genome organization of 10 segments. Sequencing coverage is shown for each position along the length of the 10 segments of strains MLBC1313 and WDBP1716; genome coding arrangements and terminal sequences are shown beneath the coverage graphs.

We performed metatranscriptomic sequencing on the third-passage BHK-21 cell suspension, which resulted in 7,660,318, 3,784,042, and 6,764,830 sequencing reads for libraries MLBC1302, MLBC1313, and WDBP1716, respectively. Full genome sequences (100%) of viruses were obtained by comparing assembled contigs against nr databases; we identified the viruses as NBV based on high sequence similarity for each segment (93.5 to 99.9%). Raw reads were subsequently mapped to draft genome sequences to correct assembly errors at 5′ and 3′ ends of the genome sequences, based on which we estimated the coverage for each genome segment, 22,383- to 36,228-fold, 23,867- to 33,225-fold, and 11,743- to 19,264-fold, respectively, for WDBP1716, MLBC1302, and MLBC1313 samples (see Table S2 in the supplemental material). We therefore named the three new strains of Nelson Bay reovirus WDBP1716, MLBC1302, and MLBC1313, respectively. The open reading frame (ORF) and coding arrangement of each segment were similar to those of other members of NBV. Except for S1, which is polycistronic, all other segments encoded only one protein (Fig. 2), with noncoding regions of about 13 to 92 bp (Table S2). The complementary conserved sequences at the 5′ and 3′ ends of the genomes were 5′-GCUUUA and UCAUC-3′. The alignment revealed high sequence homology between strains MLBC1302 and MLBC1313, ranging from 99.8% (S4) to 100% (L2), and so further analyses were of MLBC1313 as a representative in subsequent analyses.

### Evolutionary history.

**FIG 3:**
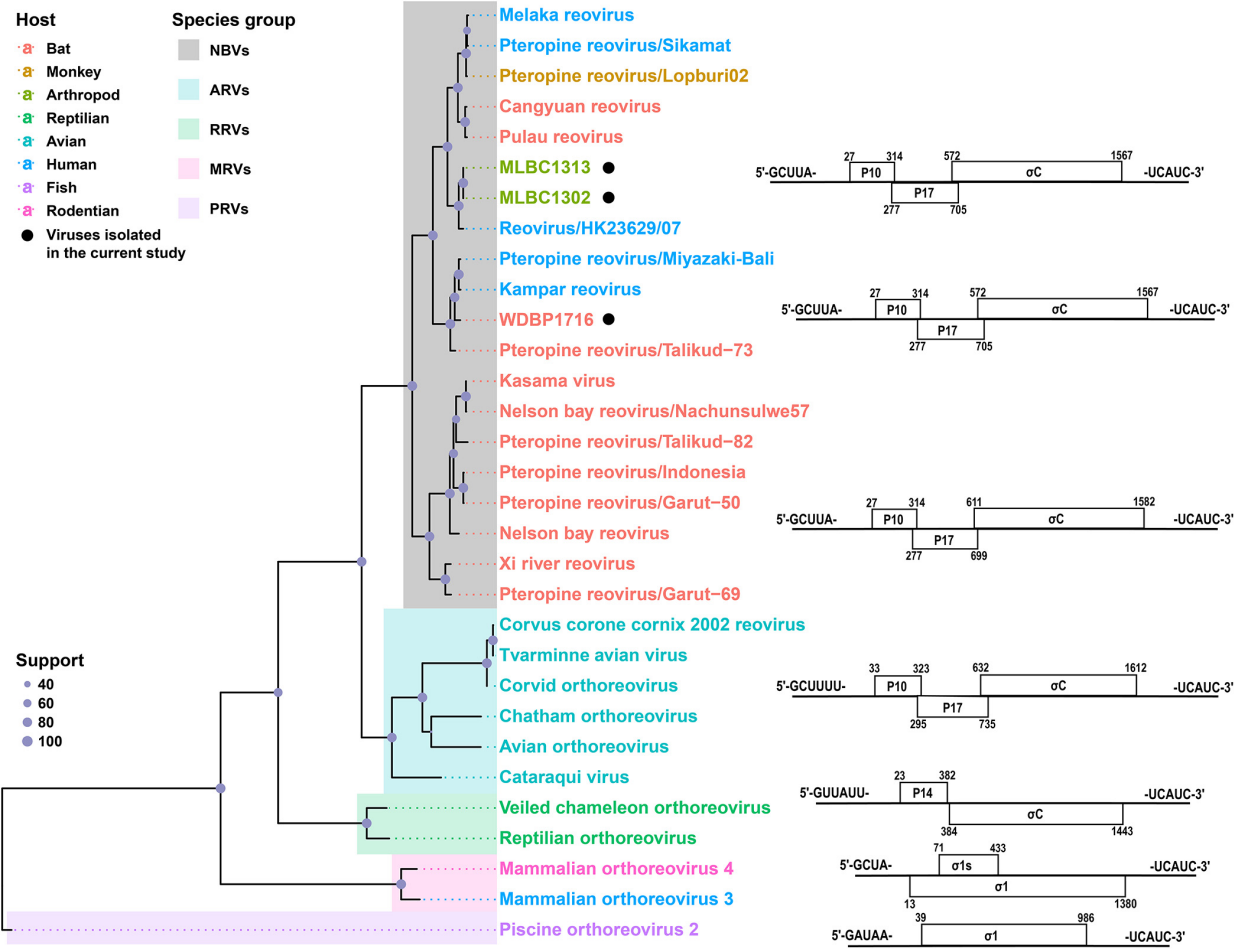
Phylogenetic trees and genome organization of S1 segment (S4 for PRV). The maximum likelihood tree was reconstructed based on the gene σC/σ1 nucleotide sequences of representative strains for the genus *Orthoreovirus*. The different colors indicate different species groups and hosts: red (bat), golden (monkey), aqua (bat fly), green (reptilian), cyan (avian), blue (human), purple (fish), pink (rodent); the different color blocks represent different species groups, and the solid black circles indicate viruses that were isolated in this study. The coding arrangements of viruses are shown behind the strain names.

We reconstructed a maximum likelihood tree based on the nucleotide sequences of a virus-cell attachment protein (σC/σ1), the most variable gene of the genus, for comparing the three novel NBV strains with the existing members of other orthoreoviruses. All three viruses were clustered within the diverse NBVs (Fig. 3). Furthermore, the S1 genome segment structure and the conserved sequences of the three viruses also followed that of NBV, suggesting that the newly discovered viruses belonged to the NBV group. They were divided into two lineages: MLBC1302 and MLBC1313, isolated from bat flies, and were determined to be closely related to HK23629/07 (89.6% identity), whereas WDBP1716 isolated from bat was closely related to Kampar orthoreovirus (86.1%). Importantly, both strains HK23629/07 and Kampar orthoreovirus were associated with human infections, which implies zoonotic potential of the newly discovered viruses (Fig. 3). Within the NBV group, both the monophyletic lineage related to bats and the complex lineages from different host species existed. Some of the human-origin strains were closely related to those isolated from bats, such as Melaka orthoreovirus and Pulau reovirus, which were all discovered in Malaysia, emphasizing the risk of NBV spillover from bats and causing diseases.

### Genomic reassortment between NBV strains.

**FIG 4:**
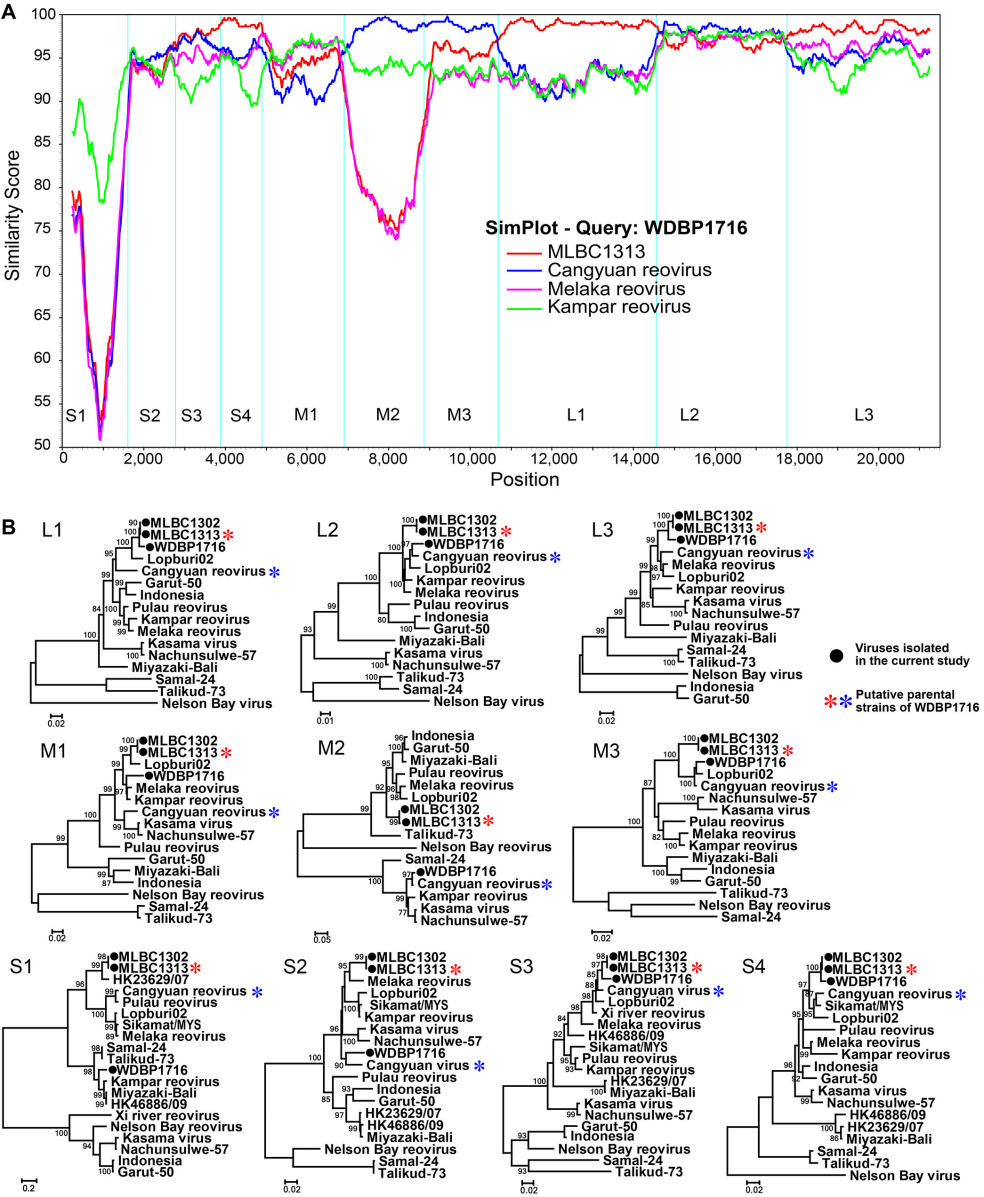
Simplot analysis for WDBP1716 and phylogenetic analysis of NBVs. (A) Similarity plot based on a concatenated genome alignment of WDBP1716. Full-length nucleotide sequences of newly isolated strain MLBC1313, Cangyuan orthoreovirus, Melaka orthoreovirus, and Kampar orthoreovirus were used as reference sequences. (B) Phylogenetic trees are based on the nucleotide sequences of 10 segments. Solid black circles and asterisks indicate newly isolated strains in the current study and the putative parental strains of WDBP1716, respectively.

To detect potential reassortment signals between different NBV strains, we performed Simplot analyses on concatenated genome segments. The consecutive nucleotide identity (a percentage) among the query and parental strains is displayed (Fig. 4A). Several crossovers occurred between Cangyuan orthoreovirus and the newly discovered strain MLBC1313: the breakpoints took place in the junctions of concatenated segments, such as M1 and M2, M3 and L1, or L2 and L3, indicating the signal of reassortment events. To further confirm these reassortment events, 10 segments of the relevant NBVs were analyzed individually by phylogenetic trees (Fig. 4B). Phylogenetic incongruence was detected between several segments, with WDBP1716 being most closely related to MLBC1313 in segments S3, S4, L1, and L3, whereas those most closely related to Cangyuan orthoreovirus were with segments S2, M2, M3, and L2, which was consistent with the result using Simplot. Therefore, we propose that strain WDBP1716 as a reassortant. Moreover, the discordant relationship between different segments is also apparent for the other strains, implying that reassortment in NBV is the norm rather than exception.

## DISCUSSION

We isolated three NBV strains from bats and their parasitized blood-sucking arthropods (bat flies) captured in Wanding Town and Mengla County near the China-Myanmar border area of Yunnan Province, and we obtained the complete sequences of the three strains from the metatranscriptomic sequencing of isolated viruses (Fig. 2). Almost all currently known viruses in the genus *Orthoreovirus* are associated with vertebrate hosts (5), which seems also decided the cluster of major groups of this genus, shown by the phylogenetic analysis (Fig. 3). For the NBVs, previous studies demonstrated that bats (7–13), monkeys (14), and humans (15–21) were corroborated to carry this virus group. The current study successfully isolated two novel NBVs from blood-sucking bat flies (*Eucampsipoda sundaica*) on the body surface of bats. To our knowledge, it is the first time that NBVs were isolated from arthropod vectors (bat flies). One possible concern is that the virus was potentially associated with blood meal rather than the bat flies. Nevertheless, the bat fly specimens were collected alive from the body surface of bats and allowed 24 h of digestion of blood meals before being placed in liquid nitrogen, which dramatically reduced the possibility that the virus was only associated with blood meals.

The previous study showed that human-origin Melaka orthoreovirus and bat-associated Pulau orthoreovirus were capable of replicating and causing syncytial CPE in a wide range of cell lines, including mosquito-derived C6/C36 (15). We attempted to grow the three isolates in C6/C36 cells; however, the absence of CPE and the decrease of virus abundance in each passage both suggested that the new viruses could not be isolated using this cell line. Similarly, strains previously isolated from bat flies (*Eucampsipoda africana*) and identified as a novel species within the genus *Orthoreovirus*, namely, Mahlapitsi virus (MAHLV), were also not capable of replicating in C6/C36 cells (36). However, these observations do not indicate that these viruses cannot replicate in insect cell lines. Indeed, since the virus was identified in bat flies, which possess a unique ecological niche with bats (37), implying they might not be compatible with growing in mosquito cell lines. Collectively, we could not rule out the possibility that bat flies may serve as potential transmission vectors of NBVs.

Our study also revealed the evolutionary history of NBV genomic segments. For nearly 20 years, at least 16 NBVs have been discovered as infecting bats and monkeys with spillover to humans, demonstrating a sizable expansion of diversity for the genus (11, 14). The phylogeny of gene σC revealed that NBVs lineages are not broadly specific with host species, implying that host-switch events may occur among different hosts (Fig. 3). The newly isolated strains WDBP1716 and MLBC1302/MLBC1313 showed the closest relationships in segments S4, L1, and L3, but the homology and phylogenies of segments M2, M3, and L2 displayed different results. Similar phylogenetic incongruence was also found between other strains, suggesting frequent reassortment events occurred during NBV coinfection. In addition, the different evolution patterns based on the ORFs of 10 segments also revealed highly divergent structural proteins, probably due to both frequent interspecies or cross-species transmission and reassortment of viruses (38), which undoubtedly further contributed to the expansion of the viral evolutionary diversity.

We also considered the zoonotic potential of the discovered viruses. Bats carry a large number of pathogens, including transmission from bats of viruses such as Nipah virus (39), severe acute respiratory syndrome coronavirus (40), and Ebola virus (41), and cause highly pathogenic diseases. Even the pathogens of the current worldwide 2019 coronavirus disease pandemic are suspected to be of bat origin (42). Previously, NBVs were not known to cause infections in humans or to be associated with respiratory or enteric diseases. Nevertheless, the idea that NBV were not pathogenic to humans was later changed by the discovery of Melaka orthoreovirus, which was isolated from patients with acute respiratory diseases, and the capability of human-to-human transmission for the virus (15). More strains have since been isolated from patients, such as Kampar orthoreovirus and Pteropine orthoreovirus (15–18), and as a result NBV is now considered a zoonotic virus in general (16–21). The two viruses isolated from this study, MLBC1302 and WDBP1716, showed high homology at virus-cell attachment protein (σC) and membrane fusion protein (P10) with Pteropine orthoreovirus HK2329/07 and Kampar orthoreovirus, respectively (Fig. 4B), which were isolated from patients with acute respiratory disease, suggesting that these viruses pose potential threats to the public health of the local residents. Therefore, an epidemiological investigation of the local population for evidence of NBV infection is needed.

In summary, we have isolated three NBV strains in the orchard in Wanding Town and Mengla County and have a more comprehensive understanding of their vector and genomic characterization, but the status of their presence in local humans and other bat populations remains unclear. Therefore, more investigations and studies should be carried out on bats and humans in more areas of Yunnan Province to provide a scientific basis for disease prevention and control measures.

## MATERIALS AND METHODS

### Ethics statement.

This research, including the procedures and protocols of specimen collection and processing, was reviewed and approved by the Medical Ethics Committee of the Yunnan Institute of Endemic Diseases Control and Prevention (file 20160002). All experiments were performed with approval by the Biosafety Committee of the Yunnan Institute of Endemic Diseases Control and Prevention. All virus isolation and nucleic acid extraction procedures were conducted in biosafety level 2 facilities, following biosafety guidelines.

### Sample collection.

Bats were captured using sticky nets in orchards of Wanding Town (longitude 98.10′E, latitude 24.08′N) and Mengla County (longitude 101.57′E, latitude 21.48′N), Yunnan Province. Bat fly specimens were collected from the body surfaces of bats using tweezers. The bats were set free after the sampling of bat flies. The collected bat flies were placed into tubes with records, transported back to the local laboratory, and left in gauze bags for up to 24 h to digest the blood meals. Then samples were stored in liquid nitrogen after classification based on morphology and transported back to the laboratory for further processing. During the sampling, we also encountered several deceased bats, and their tissues were obtained and transferred to the lab in liquid nitrogen.

### Host species identification.

The species identifications of bats and bat flies were initially performed based on morphology and subsequently confirmed with PCR and sequencing that targeted the cytochrome *c* oxidase subunit I (*COI*) gene. DNA of bat flies was extracted using QIAamp DNA minikit (Qiagen, Germany). Bat fly *COI* sequences were obtained by Sanger sequencing as previously described (43), while bat *COI* sequences were obtained by analyzing the metatranscriptomic sequencing results. The obtained sequences were subsequently compared against the BOLD database for species identification (http://www.boldsystems.org/).

### Cell culture.

Baby hamster kidney (BHK-21) and African green monkey kidney (Vero E6) cell lines were stored in liquid nitrogen in our laboratory. Minimal essential medium (MEM; Gibco, USA) containing 10% fetal bovine serum (Invitrogen, Carlsbad, USA), 1% penicillin-streptomycin combination, 1% glutamine, and 2% NaHCO_3_ (pH 7.4) was used for culturing cells in a humidified incubator at 37°C with 5% carbon dioxide (CO_2_). C6/36 Aedes albopictus cells were cultured in RMPI 1640 (Invitrogen, Carlsbad, USA) containing 10% fetal bovine serum (Invitrogen, Carlsbad, USA) and 1% penicillin-streptomycin combination, then propagated and maintained at 28°C with 5% CO_2_ (44, 45).

### Virus isolation.

Bat spleens and bat fly specimens were placed in a tissue grinder, washed with 2 mL of MEM, and homogenized with 1 mL of MEM containing 10% penicillin-streptomycin solution at low temperature. The homogenate was then centrifuged at 18,000 rpm and 4°C for 20 min, and the supernatants were inoculated into monolayer BHK-21 cells and VeroE6 cells, respectively, which were subsequently cultured in a humidified incubator at 37°C with 5% carbon dioxide after adsorbption at 37°C for 1 h. Isolation was considered successful if CPE was observed in three continuous cell passages (44, 45). After growing three passages in BHK cells, the supernatants were also inoculated into mosquito-derived cells (C6/C36) and cultured in three passages. Subsequently, virus abundance was estimated based on two genes (λB and σA) by using a quantitative RT-PCR assay. The specific primers were designed based on the sequences obtained in the current study, consisting of λB_F (CGTGCGTGCTTCCAGCTAA), λB_R (GGAGGGAGAATCGGCAACA), σA_F (GGACAGATGCTGCCTTGTCA), and σA_R (GATTCGCCCAACGTGGATA).

### Electron microscopy.

Vero E6 cells were harvested at 16 h after being infected with second-passage cell supernatant, centrifuged, and cell pellets were fixed with 2% formaldehyde and 2.5% glutaraldehyde. Polymerized samples for ultrathin (80-nm) sections were embedded using epoxy resin PON812 and stained with uranyl acetate and lead citrate. Sections were observed by electron microscopy after staining, and cells and viruses were visualized at 10,000× and 50,000× magnification (45). Then, we combined the two images together (a higher-magnification image as an inset to show details of virions) using Adobe Illustrator CC.

### Viral RNA extraction and electrophoresis.

After the culture and harvest of BHK-21 cells infected with the third-passage cell supernatant, total RNA was extracted and purified from 140 μL of the cell suspension where CPE appeared using an RNeasy Plus minikit (Qiagen, Germany). The purified RNA was separated by agarose gel electrophoresis, and genome segments were visualized by E-Gel imager (Tanon 2500B) with GoldView staining.

### RNA library construction and sequencing.

The RNA libraries were constructed by using a Zymo-Seq RiboFreem total RNA library kit (Zymo Research, USA) following the instructions provided by the manufacturer. All RNA libraries were sequenced using the Illumina Novaseq 6000 platform.

### Virus genome assembly and characterization.

For all sequencing reads, low-quality reads were removed first by using Trimmomatic (46) followed by *de novo* assembly using the Trinity program (47). The assembled contigs were then compared against the National Center for Biotechnology Information (NCBI) nonredundant protein (nr) database using DIAMOND BLASTx, with an E value threshold of 1 × 10^−5^ (48). The viral contigs associated with genome segments of orthoreovirus were then identified from the three RNA libraries, and contigs with unassembled overlaps were further merged using the SeqMan program implemented in the DNASTAR software package (Lasergene). The termini of each segment was determined by mapping against the reference sequences (NC_038658 to NC_038667, Pulau reovirus) using Bowtie2 (49). The final genome (consensus) sequences were determined by mapping the reads against draft genome sequences. Viral abundance was estimated by the number of reads mapped to genome (49). For obtained virus genomes, potential ORFs and coding arrangements were predicted using ORFfinder (https://www.ncbi.nlm.nih.gov/orffinder/) and annotated by using the online Blastp program (https://blast.ncbi.nlm.nih.gov/Blast.cgi).

### Phylogenetic analysis.

To investigate the evolutionary relationships among the novel strains with the other orthoreoviruses, reference nucleotide sequences for all segments of NBVs (see Table S3 in the supplemental material), and nucleotide sequences of virus-cell attachment protein (σC/σ1) for representative viruses of genus *Orthoreovirus* (Table S4) were downloaded from the NCBI website. MAFFT was used to align the nucleotide sequences (50), the terminal sequences were removed manually, and ambiguously aligned sequences were removed using trimAl (51). Phylogenetic trees were reconstructed using the maximum likelihood method implemented in PhyML program, with the GTR+G substitution model and SPR tree topology optimization algorithm (52).

### Genomic reassortment analysis.

The complete genome sequences for 3 viruses identified in this study and the other members of NBV downloaded from GenBank were aligned and compared. Simplot analyses were performed based on a concatenated genome alignment that used strain WDBP1716 as query and Melaka orthoreovirus, Cangyuan orthoreovirus, Kampar orthoreovirus, and MLBC1313 as parental strains (53). The analyses were based on a Kimura 2-parameter distance model, a 20-bp step size, and a 500-bp window size for comparisons. To confirm reassortment signals identified by Simplot, phylogenies were reconstructed based on the alignment of each segment and alternative clustering.

### Data availability.

All nucleotide sequences of newly isolated NBVs in this study have been deposited in GenBank (accession numbers OP913242 to OP913271).
